# Molecular characteristics of immunocytes infiltration in primary central nervous system lymphoma

**DOI:** 10.3389/fgene.2022.921823

**Published:** 2022-08-17

**Authors:** Linyun Zhang, Fei Sun, Xiaona Lu, Xiaotong Wang, Jie Wang, Jun Li, Yingsong Xu, Daqing Kou, Hongtao Lv, Bin Don

**Affiliations:** ^1^ Department of Neurosurgery, First Affiliated Hospital of Dalian Medical University, Dalian, China; ^2^ Institute of Cancer Stem Cell, Dalian Medical University, Dalian, China; ^3^ Department of Surgery, First Affiliated Hospital of Dalian Medical University, Dalian, China; ^4^ Department of Clinical Laboratory, First Affiliated Hospital of Dalian Medical University, Dalian, China

**Keywords:** primary central nervous system lymphoma, tumour immune microenvironment, network analysis, chemotherapy, immunotherapy

## Abstract

**Background:** Primary central nervous system lymphoma (PCNSL) is a rare B-cell lymphoma of central nervous system, which is often found in immunocompromised patients. The common clinical treatment of PCNSL is methotrexate (MTX) and whole brain radiation therapy. With the development of tumour immunology research, the tumour microenvironment of PCNSL is characterised by abnormal expression of different immune signature molecules and patients with PCNSL may benefit from tumour immunotherapy.

**Methods:** In our research, RNA-seq data from 82 PCNSL patients were collated by mining the microarray data from the GEO database. All samples were classified into three types related to tumour immune response by the Cibersort algorithm and consistent clustering. Differential analysis of genes was used to uncover 2 sets of differential genes associated with tumour immunity. The ICI scores of each sample were obtained by PCA algorithm, and the relationship between ICI scores and immune checkpoint expression, immunotherapy and drug sensitivity was investigated. Genes associated with ICI scores and their functional characteristics were investigated by WGCNA analysis and PPI analysis, based on the ICI scores of each sample.

**Results:** The tumour microenvironment in PCNSL has a greater relationship with the tumour immune response. ICI scores obtained from 375 differential genes were associated with multiple immune responses in PCNSL. PCNSL patients with higher ICI scores had a better tumour microenvironment and were sensitive to immunotherapy and some small molecule drug. This study also identified 64 genes associated with ICI scores, which may serve as important therapeutic and prognostic targets for PCNSL.

**Conclusion:** The presence of multiple immunosuppressive responses in the tumour microenvironment of PCNSL which suggested that improving the immune function of PCNSL patients through immunotherapy and targeted therapies can be an effective treatment for PCNSL. And the ICI score and associated genes may also provide a better predictor of the clinical use of immunotherapy.

## Introduction

Primary central nervous system lymphoma (PCNSL) is an uncommon but extremely aggressive form of extranodal non-Hodgkin’s lymphoma, accounting for 4–6% of all extranodal lymphomas, 1% of all lymphomas and approximately 2% of all central nervous system tumours (Kluin, 2008). It is a very aggressive malignancy that affects just the craniospinal axis and has no systemic spread (brain > eye > molluscum contagiosum > spinal cord). Histologically, more than 90% of cases are categorized as diffuse large B-cell lymphoma (DLBCL) (Grommes and DeAngelis, 2017), almost half of patients have R/R PCNSL (refractory or recurrent PCNSL), and over 50% of patients are between 60 and 80 years of age (Mendez et al., 2018). Recent studies have shown an increase in the overall incidence of PCNSL, with 5- and 10-years survival rates of 29.9 and 22.2%, respectively, (Ostrom et al., 2015). Immune dysfunction is the sole recognized risk factor (Bathla and Hegde, 2016) and an increased frequency of PCNSL has been observed in individuals with acquired immunodeficiency (acquired immunodeficiency syndrome or post-transplant disease) and congenital immunodeficiency (X-linked lymphoid hyperplasia syndrome, Wiskott-Aldrich syndrome or ataxia capillaris) (Erdag et al., 2001). PCNSL presents clinically in a non-specific manner, with the most prevalent symptoms being cognitive decline and gait abnormalities (Izquierdo et al., 2016).

Over the past several decades, treatment for people with PCNSL has improved considerably. The median overall survival of elderly patients aged 50–69 years has been reported to have increased from 8 months in the 1970s to 35 months in the 2010s (Mendez et al., 2018). Chemotherapy, radiation, haematopoietic stem cell transplantation, and new targeted medicines are all considered salvage treatments (Soussain et al., 2001; Sierra del Rio et al., 2009; Pentsova et al., 2014; Atilla et al., 2019). 3-years survival rates of 53% and OS of 64% have been achieved in patients with R/R PCNSL, usually using high-dose chemotherapy and autologous haematopoietic stem cell transplantation (Soussain et al., 2001). For newly diagnosed PCNSL, high-dose methotrexate (HD-MTX)-based regimens are utilized as first-line therapy, however the best effective dosage of HD-MTX has yet to be found. Rituximab has been shown to be effective in improving clinical outcomes in systemic lymphoma and the efficacy of single agent rituximab has been reported in patients with R/R PCNSL (Batchelor et al., 2011). In comparison to HD-MTX alone, combining HD-MTX with different chemotherapeutic drugs has recently been demonstrated to enhance therapy response. The combinations currently used are 1) methotrexate, temozolomide and rituximab; 2) rituximab, methotrexate, procarbazine and vincristine; and 3) methotrexate, cytarabine, rituximab and tiotipine. Immune checkpoint inhibitors might be another viable treatment option for PCNSL. Anti-PD1 monoclonal antibodies have demonstrated remarkable therapeutic effect in CNS lymphomas by inhibiting immune checkpoints (Qiu et al., 2018). There is currently no agreement on how to treat R/R PCNSL, and patients with R/R PCNSL have a poor overall survival rate (Pentsova et al., 2014; Löw and Batchelor, 2018). New alternative therapies are imperative. The purpose of this work was to establish a technique for identifying and quantifying the molecular characteristics of immunocytes infiltration (ICI) in PCNSL, with the hope of laying the groundwork for improved chemotherapy and immunotherapy for PCNSL.

## Materials and methods

### Data collection and processing

In this study, we downloaded the datasets (GSE34771, GSE155398, GSE61578 and GSE11392) related to primary central nervous system lymphoma (Primary central nervous system lymphoma, PCNSL) from the GEO database, and retained the PCNSL samples in each dataset. The “removeBatchEffect” technique was used to mitigate the possibility of batch effects across datasets. Ultimately, we obtained a comprehensive PCNSL expression matrix consisting of 82 samples and 11,743 genes. PCA analysis was utilized to represent the batch-effects before and after going to removeBatchEffect.

### Multiscale clustering of geometrical network in PCNSL

In order to analyze the complex co-regulatory relationship among genes, the co-expression network analysis of the expression matrix was carried out by using multi-scale embedded gene co-expression network analysis (MEGENA). The two key elements of this technology are multi-scale clustering structures detection and parallelization of embedded network creation. Specifically, the Pearson correlation was first utilized to compute the correlation among genes, and then planar filtered network (PFN) was implemented to screen gene pairs with significant correlation. PFN was then subjected to unsupervised clustering in order to discover network clusters (i.e., gene modules) at different compactness resolutions using multi-scale clustering analysis (MCA). The generated gene modules were arranged hierarchically, resembling a multiscale arrangement of gene modules with varying degrees of compactness. The hierarchy represented a succession of higher-order connections (i.e., parent) modules that include children modules. The connectedness is an important indicator to evaluate the nodes in parent-children undirected network. MEGENA determined smaller kid modules inside larger parent modules. The nodes with considerably (*p* < 0.05) more network connectedness than the randomly permuted planar networks are further identified as candidate main drivers of gene modules. The enriched gene ontology (GO) keywords are then used to identify the gene modules.

### Estimating of immune cell infiltration and sample clustering

To estimate 22 different forms of ICI for each patient with PCNSL, the R software “CIBERSORT” was used. Correlation analysis was performed between the ICI components. Following that, the R package “ConsensusClusterPlus” was used to do hierarchical agglomerative clustering according to the ICI pattern. “ConsensusClusterPlus” uses an algorithm to assess cluster count and membership based on unsupervised analysis evidence of stability. To guarantee clustering stability, this procedure was run 1,000 times.

### Differentially expressed genes screening and competing endogenous RNAs network construction

To discover DEGs associated with ICI, we grouped all data into distinct ICI subtypes. We investigated the DEGs between ICI subtypes with the help of the “Limma” R package (|log2foldchange|> 1, false discovery rate (FDR) < 0.05). In order to explore the possible biological functions and pathways of DEGs, “clusterProfiler” package in R was used to carry out GO and KEGG enrichment analyses. In addition, miRNet, miRTarBase and StarBase databases were utilized to predict the miRNAs of DEGs, and then miRNet, StarBase and mircode databases were used to predict the targeted lncRNAs of above miRNAs, so as to construct a comprehensive mRNA-miRNA-lncRNA regulatory network (i.e., ceRNA network) closely related to ICI pattern.

### Clustering with DEGs, dimension reduction and construction of ICI score

Unsupervised clustering was used to cluster data at the genomic level based on the expression pattern of DEGs. Notably, DEGs that linked favorably or negatively with gene clusters were designated as ICI gene signatures A and B, respectively. To eliminate noise or redundant genes, the dimensions of ICI gene signatures A and B were reduced using the Boruta method, and feature genes were discovered. Following that, principal component analysis (PCA) was used to extract the major component 1 from feature genes and assign it to the relevant ICI gene signature as the signature score. Finally, an approach comparable to the gene expression grade index was used to calculate each patient’s ICI score: ICI score = ∑PCA1A-∑PCA1B.

### Association of ICI scores with immune characteristics of PCNSL patients and therapeutic options

High- and low-ICI score groups (HSG and LSG, respectively) were defined according to the median ICI scores. In order to explore the association between ICI score and PCNSL immune characteristics, and provide clinical guidelines for therapeutic options of PCNSL patients, we carried out the following series of analyses: Gene Set Enrichment Analysis (GSEA), Gene Set Variation Analysis (GSVA), immune checkpoint molecule analysis, Tumor Immune Dysfunction and Exclusion (TIDE) analysis and chemosensitivity analysis.

With the help of “limma” package in R, we analyzed the difference of gene expression between HSG and LSG, calculated the logarithmic fold change (logFC), and sorted the logFC from big to small. Then GSEA was carried out to analyze which signal pathways were involved in HSG and LSG grouping. The reference gene sets were “C5:GO gene sets” and “C2:KEGG gene sets” in the MSigDB database (http://www.gsea-msigdb.org/gsea/).

GSVA is a non-parametric and unsupervised algorithm. Unlike GSEA, GSVA does not require group samples in advance and can calculate the enrichment scores of specific gene sets in each sample. In other words, GSVA transforms gene expression data from a single gene as a feature expression matrix to a specific gene set as a feature expression matrix. GSVA quantifies the results of gene enrichment, which is more convenient for follow-up statistical analysis. Then, based on the grouping information, the differences of GSVA results are analyzed, and the gene sets with significant differences among samples can be found. Compared with genes, these differentially expressed gene sets are more biological and explainable.

In this study, the GSVA package in R was used to analyze the gene variation based on the expression of all genes in 82 samples (the reference gene set is C2:KEGGgenesets in MSigDB database). A total of 186 signal pathways were obtained. The limma package in R is used to analyze the difference of GSVA results between HSG and LSG. The FDR <0.05 is used as the threshold to screen the signal pathways with significant differences. The correlation between significant difference signal pathway and ICI score was analyzed and visualized with the help of “ggcor” package in R.

Through literature review and data integration, we found 35 recognized immune checkpoint molecules with complete expression information in PCNSL, and then we compared the differential expression of immune checkpoint molecules in HSG and LSG subgroups through the “wilcox.test” function in R.

Tumor Immune Dysfunction and Exclusion (TIDE) is a computational approach for assessing the probability of tumor immune escape based on the gene expression profiles of tumor samples. The potential response to immunotherapy was inferred from the TIDE score, and the lower TIDE indicated that the patient responded better to immunotherapy. We imported the standardized expression data into the TIDE website (http://tide.dfci.harvard.edu/) and obtained the TIDE scores of 82 PCNSL patients. We compared the difference of TIDE scores between HSG and LSG subgroups, and analyzed the correlation between ICIscore and TIDEscore in order to explore the potential possibility of immunotherapy benefit for PCNSL patients under different ICI patterns.

To assess treatment sensitivity, the R package “pRRophetic” was used to compute the concentration producing a 50% decrease in growth (IC50) of specific inhibitors. The “wilcox.test” function in R was utilized to compute the IC50 difference between HSG and LSG. Finally, only the top 10 chemotherapeutic drugs with the most significant difference in IC50 between the two groups were retained.

### Weighted gene co-expression network analysis identification of the hub genes associated with ICI scores

Weighted gene co-expression network analysis (WGCNA) was carried out to determine hub genes significantly associated with ICI scores using the R package “WGCNA”. Pearson’s correlation analysis was used to generate an adjacency matrix. By estimating the soft-thresholding value, we developed a scale-free co-expression network. To conduct a thorough analysis of the functional modules, the adjacency matrix was transformed into a topological overlap matrix (TOM) and the dissimilarity matrix between genes was produced (dissTOM = 1-TOM). When dissTOM was clustered hierarchically, genes with comparable expression patterns were grouped together in the same gene module. A minimum of 30 module genes was specified. To acquire the gene modules, the DynamicTreeCut method was used, and modules with a high degree of similarity were further combined.

To find modules associated with ICI scores, module eigengenes (MEs) and gene significance (GS) were used. MEs were believed to be the primary component of each gene module, and ME expression was acknowledged on behalf of all genes included inside a particular module. Correlations between ICI scores and MEs were calculated in order to identify modules of clinical value. Additionally, GS was rated as the mediating *p* value for each gene in the linear regression analysis of clinical features and gene expression patterns (GS = lgP). Module significance (MS) was subsequently rated as the average GS of all genes included inside a module. The clinically important module was determined as the one with the highest absolute MS.

Each gene module membership (MM) was determined in the hub module, which served as a proxy for the module’s relevance. Genes with |GS| more than 0.65 and |MM| greater than 0.8 were deemed potential hub genes. Following that, we uploaded the aforementioned putative hub genes to the STRING database (https://string-db.org/) to develop a protein-protein interaction (PPI) network, and the core interaction was extracted using MCODE in Cytoscape. Finally, the genes in the top1 module were designated as PCNSL’s hub genes.

Considering that these hub genes were derived from the same module in WGCNA, we applied Spearman test to verify the correlation between these hub genes. In addition, we analyzed the differences in the expression of these hub genes among different ICI subtypes and their correlation with PCNSL immune microenvironment.

### Hub gene-based ceRNA network establishment

The target miRNAs of hub genes were predicted by using miRNet, miRTarBase and StarBase databases, respectively, and the mRNA-miRNA relation pairs were intersected from the three databases. The target lncRNAs of the above miRNAs were then predicted by using miRNet, StarBase and mircode databases, respectively, and the miRNA-lncRNA relation pairs obtained from the three databases were also intersected. Finally, according to the shared miRNA, the mRNA-miRNA-lncRNA relationship was combined to plot the ceRNA network of the hub genes.

### Statistical analyses

R software (version 4.1.0) and cytoscape software (version 3.9.2) was employed for statistical analyses and network plots separately. The R packages “MEGENA (version 1.3.7)”, “clusterProfiler (version 4.0.2)”, “ConsensusClusterPlus (version 1.40.0)”, “corrplot (version 0.90)”, “limma (version 3.48.3)”, “stats (version 3.48.3)”, “ggcor (version 0.9.8.1)”, “pRRophetic (version 1.4.0)”, “GSVA (version 1.40.1)”, “WGCNA (version 1.70–3)”, “ggplot2 (version 3.3.5)”, and “psych (version 2.1.6)” were utilized in our study. The specific prameter settings of MEGENA were as follows: n. cores = 2, method = “pearson”, FDR. cutoff = 0.05, module. pval = 0.05, hub. pval = 0.05, cor. perm = 10, hub. perm = 100. Unsupervised clustering parameter settings were as follows: reps = 1,000, pItem = 0.8, clusterAlg = “pam”, distance = "pearson”, innerLinkage = "complete”. Default parameter settings of cytoscape were as follows: Degree Cutoff: 2, Node Socre Cutoff: 0.2, K-Core: 2, Max. Depth: 100.

For comparisons between two groups, the Wilcoxon test was used, while the Kruskal–Wallis test was used for comparisons between more than two groups. To obtain the correlation coefficient, a Spearman analysis was used. The significance level was set at P 0.05.

## Results

### Multiscale clustering of geometrical network in PCNSL

**FIGURE 1:**
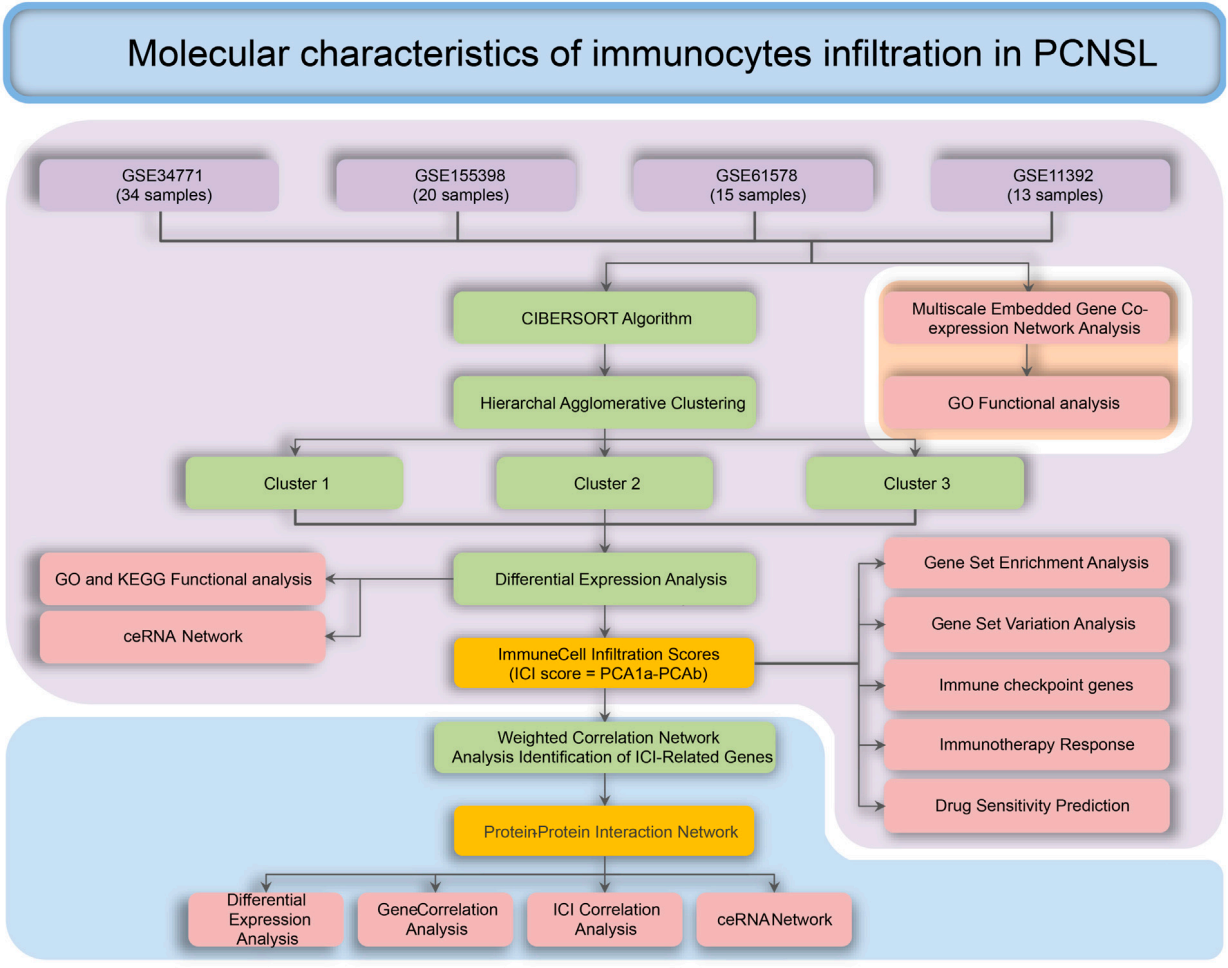
Process for the analysis of the molecular characteristics of immune infiltrating cells in patients with PCNSL.

**FIGURE 2:**
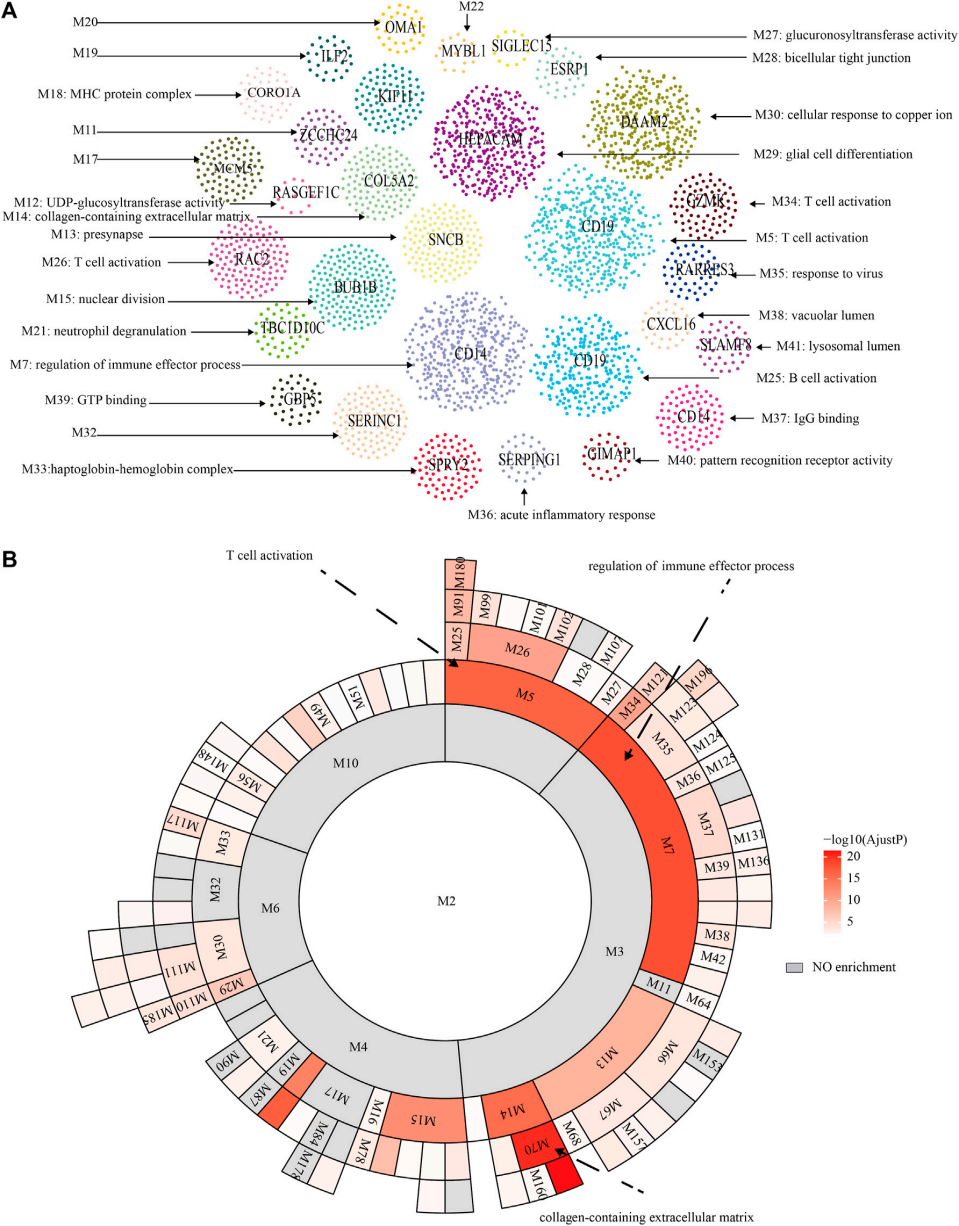
MEGENA analysis. **(A)** Functional modules of MEGENA analysis. **(B)** Hierarchy of functional modules of MEGENA analysis.

The flowchart of the whole study was presented in Figure 1. After data collection and processing, a total of 82 samples accompanied by the sequencing results of 11,743 genes were obtained (Supplementary Table S1). The PCA analysis before and after remove of batch effect is shown in Supplementary Figure S1. The results of the PCA analysis showed significant grouping between original samples and insignificant grouping between samples after removal of batch effects. To further analyze the intricate co-regulation connection between the genes in PCNSL, we used MEGENA to conduct a co-expression network analysis on the 11,743 genes expressed in 82 human PCNSL samples. From the co-expressed gene network, we identified 119 gene modules with a hierarchical structure, showing parent-child relationships (Supplementary Tables S2, S3). The topmost modules in the network are assigned at a particular compactness scale (Figure 2A). The module hierarchical structure and the enriched BP terms are also summarized in Figure 2B. Module 5 (M5), M26, and M34 are most significantly enriched for T cell activation. M18, M21, M7, M35, M25, M37, M40, and M36 are most significantly enriched for MHC protein complex, neutrophil degranulation, regulation of immune effector process, response to virus, B cell activation, IgG binding, pattern recognition receptor activity, acute inflammatory response, respectively, suggesting that immune response plays a crucial role in the pathophysiological processes of PCNSL.

### The landscape of ICI in PCNSL

**FIGURE 3:**
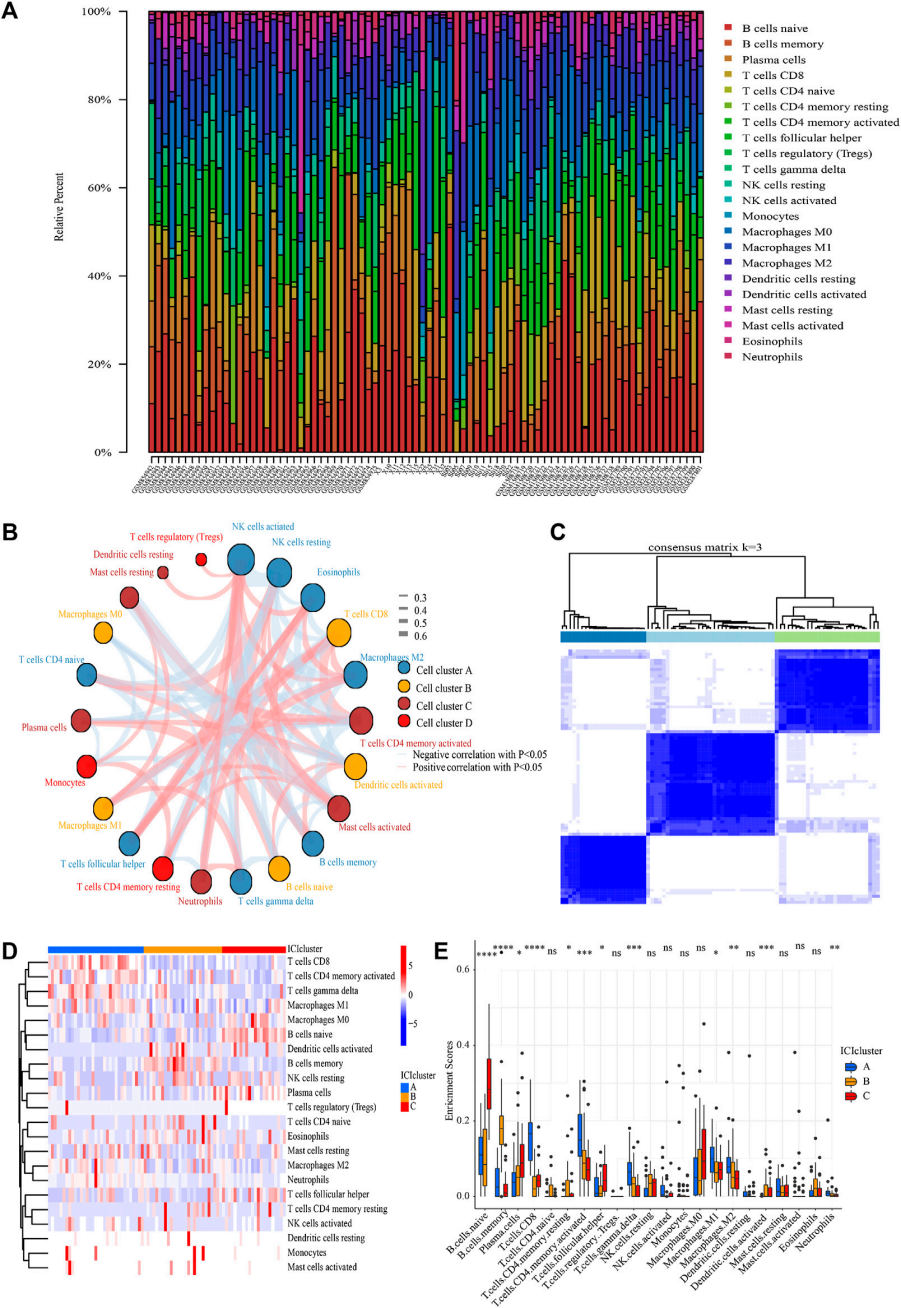
ICI analysis and sample clustering. **(A)** Relative proportions of 22 immune cell subpopulation in PCNSL samples. **(B)** Cellular interaction of 22 immune cell subpopulation. **(C)** Consensus matrixes of all PCNSL samples for appropriate k value (*k* = 3). **(D)** The heat map showed unsupervised clustering of 22 immune cell subpopulation in all PCNSL samples (Rows represented tumor-infiltrating immune cells, and columns represented samples). **(E)** The fraction of tumor-infiltrating immune cells in three ICI clusters (The Kruskal–Wallis test was used to assess the statistical difference among three ICI clusters).

The landscape of ICI was systematically evaluated by CIBERSOFT algorithm. Figures 3A,B summarizes the abundance of ICI in each PCNSL sample and the interaction of immune cells. Obviously, the proportions of ICI in PCNSL varies significantly and there are significant correlations among these immune cells, suggesting that significant heterogeneity is observed for immune microenvironment of PCHSL. Thus, classifying PCNSL immunophenotypes based on the distinct traits of ICI would therefore be highly warranted.

Following that, all samples were clustered using the R package “ConsensusClusterPlus” based on their ICI content, and three subtypes were identified (Figure 3C). To more precisely define the intrinsic distinctions between the three ICI subtypes, a differential study of immune cells in the three ICI subtypes was performed (Figures 3D,E). The ICI cluster A was accompanied by high CD8+T cells, activated memory CD4+T cells, M1 macrophages, γδT cells, M2 macrophages, neutrophils, as well as low naïve B cells, memory B cells, resting natural killer (NK) cells, activated dendritic (DC) cells, and resting memory CD4+T cells. However, the opposite was the case for ICI cluster B, which was accompanied by high memory B cells, naïve CD4+T cells, as well as low CD8+T cells, activated memory CD4+T cells, M1 macrophages, γδT cells, and neutrophils. The ICI cluster C was marked by high naïve B cells, plasma cells, follicular helper T (Tfh) cells, as well as low CD8+T cells, activated memory CD4+T cells, M1 macrophages, γδT cells, memory B cells, naïve CD4+T cells. Thus, cluster A of PCNSL patients showed active T lymphoid system response, cluster C of PCNSL patients showed active B lymphoid system response, and cluster B of PCNSL patients showed inactive immune response.

### Differentially expressed genes screening and competing endogenous RNAs network construction

**FIGURE 4:**
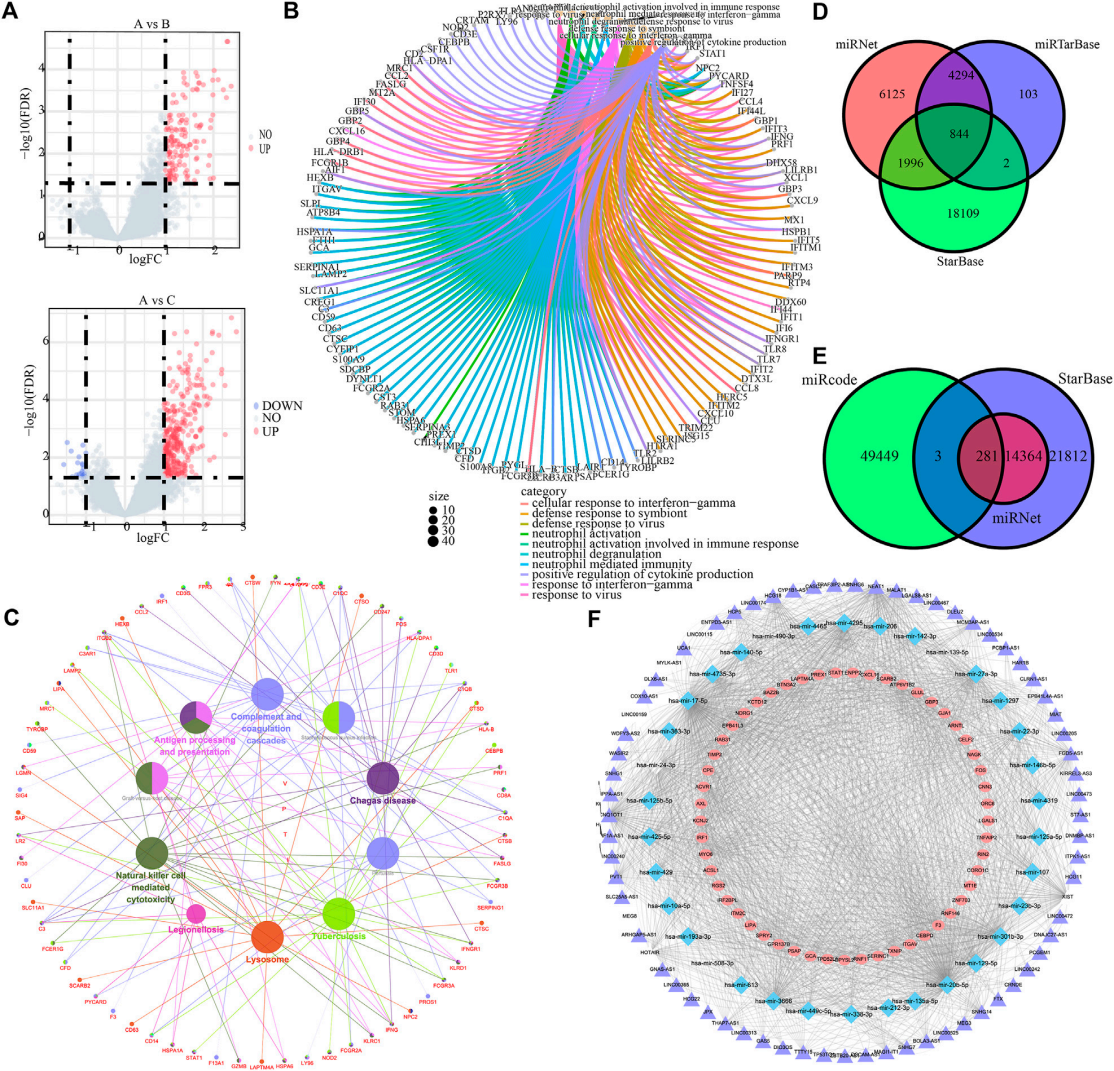
ICI differential genes and associated ceRNAs. **(A)** Volcano maps based on the distribution of all genes in clusterA and clusterB, clusterA and clusterC. **(B)** GO enrichment analysis for the top 10 most significant biological functions. **(C)** Cytoscape differential gene KEGG enrichment analysis. **(D)** mrna-mirna relationship pairs predicted by miRNet, miRTarBase and StarBase databases. **(E)** Predicted lncRNAs from miRNet, StarBase and mircode databases. **(F)** mrna-mirna-lncrna regulatory network.

To prepare for the development of ICI scores and illustrate the heat map of DEGs between ICI subtypes, we utilized the R package “Limma” to analyze the DEGs between the three ICI subtypes at the genomic level, yielding a total of 375 DEGs (Figure 4A, Supplementary Tables S4, S5). GO and KEGG results showed that these DEGs were mainly enriched in neutrophil-related immune response, interferon-γ-related cellular response, regulation of cytokine production, antigen processing and presentation, complement cascades, and NK cell-mediated cytotoxicity (Figures 4B,C).

Subsequently, miRNet, miRTarBase, and StarBase databases were employed to predict the target miRNAs of 375 DEGs, and 844 pairs of mRNA and miRNA interaction involving 141 mRNAs and 312 miRNAs were preserved (Figure 4D, Supplementary Tables S6–S9). Besides, miRNet, StarBase and miRcode databases were then applied to predict the target lncRNAs of 312 miRNAs, and 281 pairs of miRNA and lncRNA interaction involving 34 miRNAs and 71 lncRNAs were preserved (Figure 4E, Supplementary Tables S10–S13). Finally, 938 regulatory relations of mRNA-miRNA-lncRNA involving 53 mRNAs, 34 miRNAs, and 71 lncRNAs were obtained and visualized (Figure 4F).

### Clustering by DEGs from ICI subtypes and construction of ICI score

**FIGURE 5:**
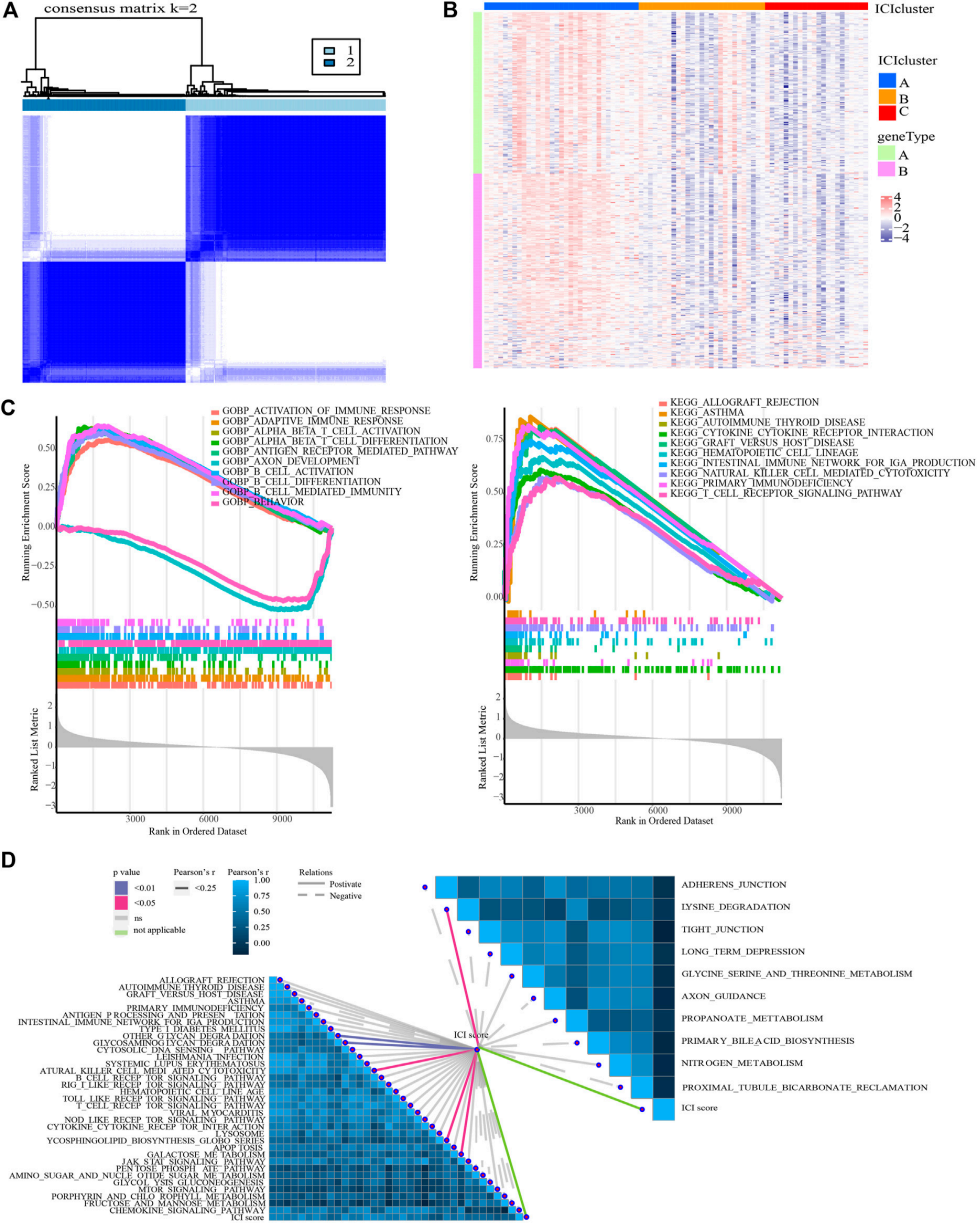
Identification of ICI gene clusters and ICI scores. **(A)** Consensus clustering matrix for *k* = 2 in PCNSL samples based on DEGs among ICI subtypes. **(B)** Expression traits of DEGs in different ICI clusters and gene clusters. **(C)** GSEA for the enrichment results in high and low ICI score groups (ICI scores = PCA1a-PCA1b). **(D)** Correlation between ICI score and typical immune-related signaling pathways.

The PCNSL samples were re-divided into two gene groups using unsupervised clustering of these DEGs (Figure 5A). The DEGs that are positively associated with gene clusters are designated as gene signature A, while the other DEGs are designated as gene signature B. Heat map visualization of the gene expression of gene signature A and B was shown in (Figure 5B).

To quantify the ICI landscape of patients with PCNSL and assist the identification of significant genes, PCA was used to calculate the aggregate score of feature genes from gene signature A and B, respectively, after dimensionality reduction. We calculated the sum of the scores and designated them as ICI scores (Supplementary Table S14). All PCNSL patients were classified into two groups based on their median ICI score: those with high ICI scores and those with low ICI scores (i.e., HSG and LSG).

### Association of ICI scores with immune characteristics of PCNSL patients and therapeutic options

GO- and KEGG-related GSEA revealed that T cell-mediated immune response, B cell-mediated immune response, and NK cell-mediated immune response were significantly enriched in the HSG subgroup (Figure 5C). In addition, GSVA results demonstrated that there were 44 signaling pathways (involving 34 up-regulated and 10 down-regulated pathways) with significant differences between HSG and LSG subgroups (Supplementary Table S15). More importantly, ICI score was detected to be significantly associated with glycan degradation, glycosaminoglycan degradation, lysine degradation, glycosphingolipid biosynthesis, galactose metabolism, and NK cell-mediated cytotoxicity (Figure 5D).

**FIGURE 6:**
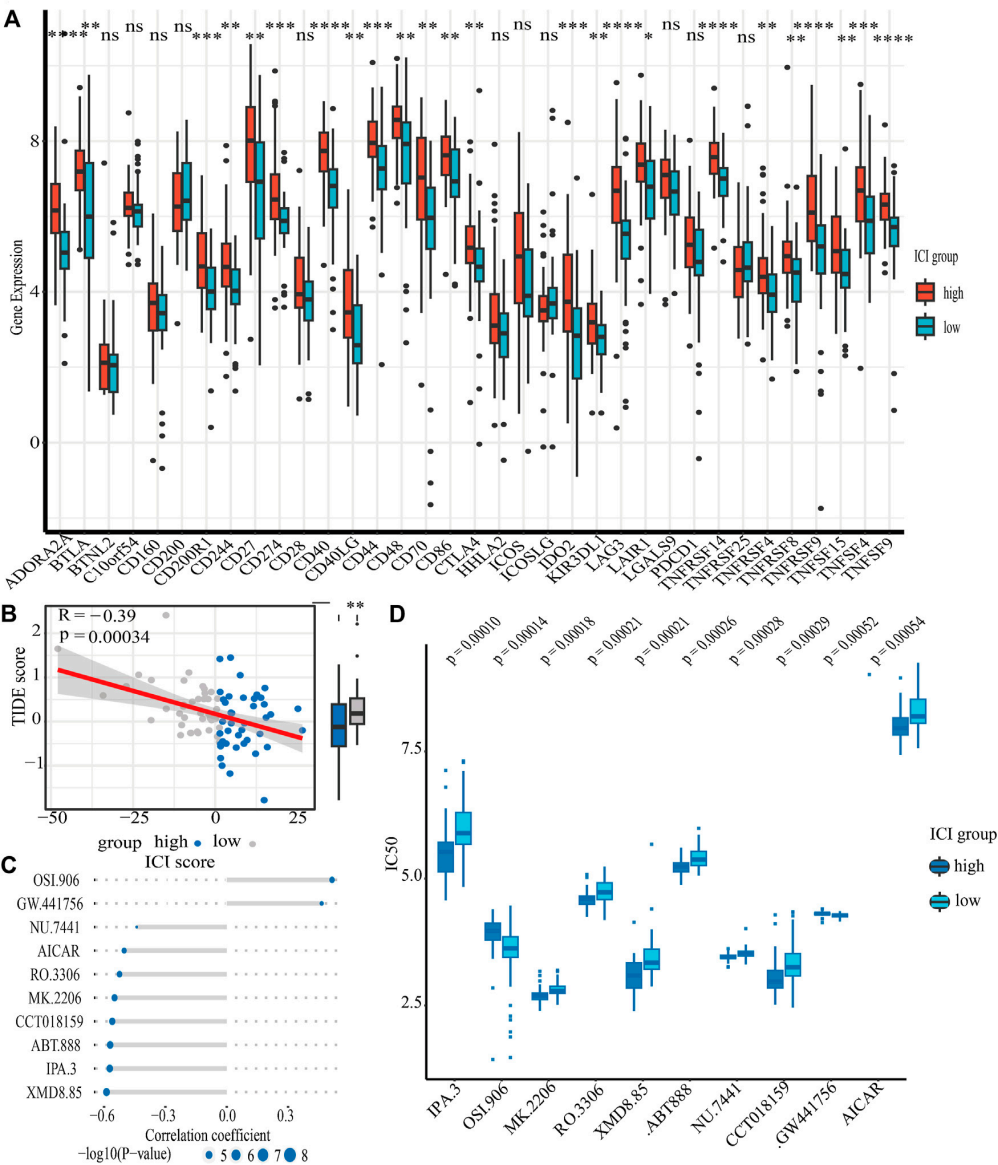
The relationship between ICI scores and drug therapy. **(A)** The results of differential expression of immune checkpoint genes in high and low ICI score groups. **(B)** The results of spearman’s correlation analysis of ICI scores with TIDE scores (TIDE scores serve as a well-recognized indicator of immunotheraptic response). **(C)** The results of spearman’s correlation analysis of ICI scores with ten targeted drugs. **(D)** The results of differential drug response analysis of ten targeted drugs in high and low ICI score groups (Lower values on the *y*-axis of boxplots imply greater drug sensitivity).

In addition to these, the expression of immune checkpoints in the HSG subgroup generally showed a high trend, suggesting that the high-risk populations might benefit from immunotherapy (Figure 6A). Subsequently, we conducted a TIDE analysis, and the results showed that the high-risk group had lower TIDE populations, indicating that the PCNSL patients in the high-risk subgroup were indeed adaptive to immunotherapy (Figure 6B).

Chemotherapy is one of the first-line treatment strategies for PCNSL patients. Due to the obvious heterogeneity of immune microenvironment in PCNSL patients, people with different immune characteristics may benefit from different chemotherapeutic drugs. Our data revealed that LSG populations might benefit from OSI.906 (a selective inhibitor of IGF-1R/IR kinase) and GW.441,756 (a selective inhibitor of the NGF receptor tyrosine kinase A); however, HSG populations might benefit from IPA.3 (a non ATP-competitive, allosteric inhibitor of p21-activated kinase 1), MK.2206 (a selective allosteric Akt inhibitor), RO.3306 (an ATP-competitive and selective CDK1 inhibitor), XMD8.85 (a selective inhibitor of ERK5 and LRRK2), ABT.888 (an inhibitor of PARP-1 and PARP-2), NU.7441 (a selective DNA-dependent protein kinase inhibitor), CCT018159 (a novel inhibitor of heat shock protein 90 with potential anticancer activity), and AICAR (a cell-permeable, allosteric activator of AMP-activated protein kinase) (Figures 6C,D).

### WGCNA identification of key genes based on ICI score

**FIGURE 7:**
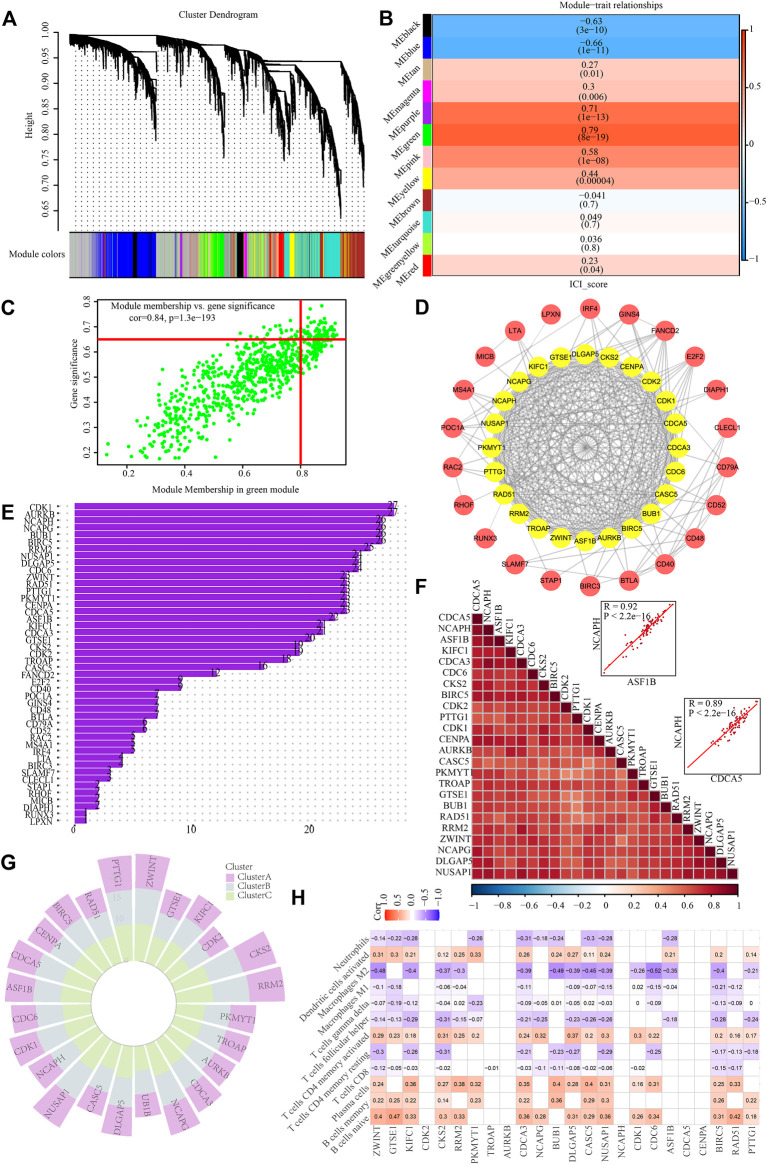
Identification of hub genes based on ICI scores. **(A)** Identification of co-expression modules for WGCNA analysis. **(B)** Correlation heat map of modules and differentially immuno-infiltrated cells. **(C)** Green module screening for core genes. **(D)** PPI network analysis. **(E)** protein counts of PPI analysis. **(F)** Hub gene correlation. **(G)** Expression traits of hub genes in different ICI subgroups. **(H)** Correlation between hub genes and immune cell infiltration in PCNSL.

To discover critical genes in PCNSL, a gene co-expression network was constructed using WCGNA to identify significant gene modules associated with the ICI score. By choosing 10 as the suitable soft threshold (Supplementary Figure S1), a scale-free co-expression network was constructed, yielding 11 modules (Figure 7A). The green module was found to have the strongest association with the ICI score (Correlation coefficient = 0.79, *p* = 8e-19) (Figure 7B). On the basis of established criteria (|GS| >0.65 and |MM| >0.8), we identified 64 highly connected genes in the salmon module as possible hub genes (Figure 7C). Following that, we performed a PPI network analysis on these 64 hub genes by uploading them to the STRING database, and 24 hub genes were discovered (Figures 7D,E). Spearman test was applied to verify their co-expressed relationship, with the data showing that there were strong positive correlations between these 24 genes (Figure 7F). In addition, we intensively explored the expression traits of these 24 genes in different ICI subgroups, and investigated the correlation between these 24 genes and 12 immune cells with significant difference among ICI subgroups (Figures 7G,H).

### Hub gene-based ceRNA network establishment

**FIGURE 8:**
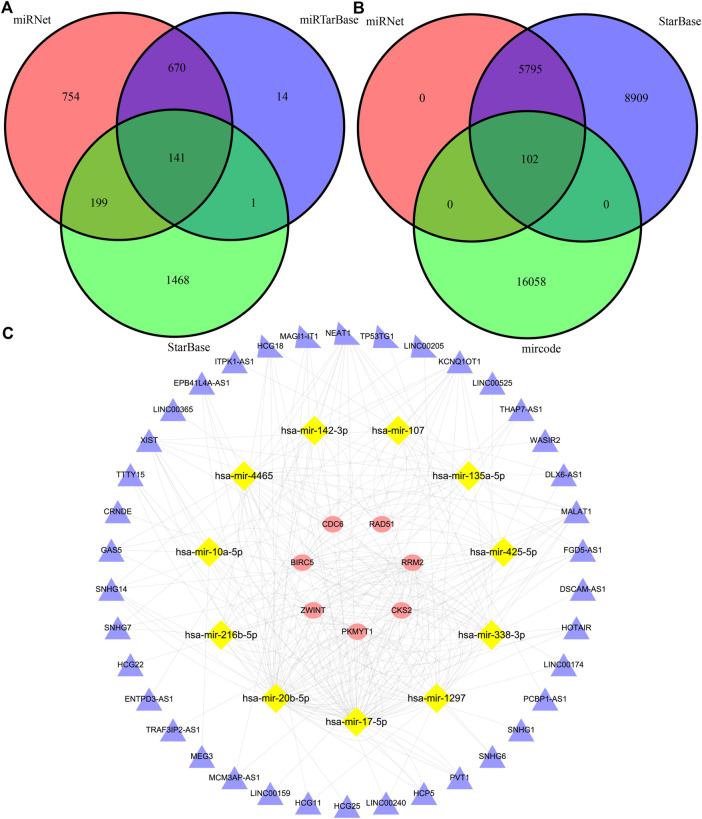
The construction of a triple-regulatory network of mRNA-miRNA-lncRNA. **(A)** miRNAs predicted by miRNet, miRTarBase and StarBase databases. **(B)** lncRNAs predicted by miRNet, StarBase and mircode databases. **(C)** mrna-mirna-lncrna regulatory network.

The miRNet, miRTarBase, and StarBase databases were employed to predict the target miRNAs of 24 hub genes, and 141 pairs of mRNA and miRNA interaction involving 16 mRNAs and 120 miRNAs were preserved (Figure 8A, Supplementary Tables S16–S19). Besides, miRNet, StarBase and miRcode databases were then applied to predict the target lncRNAs of 120 miRNAs, and 102 pairs of miRNA and lncRNA interaction involving 11 miRNAs and 38 lncRNAs were preserved (Figure 8B, Supplementary Tables S20–S23). Finally, 130 regulatory relations of mRNA-miRNA-lncRNA involving 7 hub genes, 11 miRNAs, and 38 lncRNAs were obtained and visualized (Figure 8C).

## Discussion

PCNSL is an uncommon kind of non-lymphoma Hodgkin’s that originates in the central nervous system, which is mostly high-grade B-cell lymphoma. This lymphoma frequently occurs in immunocompromised patients, while immunocompetent patients usually have a higher age of onset. The disease is commonly detected and diagnosed by cerebrospinal fluid (CSF) pathology and brain biopsy. Due to the relative rarity of the disease and the lack of prospective and randomised clinical trials, few of effective therapeutic targets and optimal treatment options can be used. As we all know the central nervous system is protected in the blood-brain barrier, traditional anatomical and physiological studies suggest that the central nervous system may be an immune tolerant organ and that its internal T and B cells are generally suppressed by the microenvironment of brain tissue. This particular microenvironment makes the pathogenesis of PCNSL is more closely related to the immune system and immune response.

PCNSL is more associated with immune cell infiltration of the microenvironment. CSF analysis used to detect the level of immune cells in the CSF is an important test for confirming diagnosis. It was found that the baseline of tumour immune response in PCNSL patients was reduced compared to the normal, and in most cases the CSF tests revealed a decrease in CD8^+^ T cells and helper DC cells, as well as a decrease in Th1 cells and an increase in Th2 cells, suggesting that the microenvironment of PCNSL inhibits the activation of most immune cells (Chang et al., 2015; Takashima et al., 2019a; Monabati et al., 2020). And there was also a degree of reduction in HLA on the surface of tumour cells in PCNSL, such as HLA-DM, which prevented immune cells from recognising the surface antigens of tumour cells (Nijland et al., 2017). In our research, the Cibersort algorithm revealed significant changes in immune cells such as B cells, T cells and macrophages in PCNSL tissues, which confirmed the presence of a strong immunosuppression in the microenvironment of PCNSL.

IL10 and IL6 are important protective cytokines which were secreted after inflammation and immune responses, which reduce cellular damage and promote the cellular repair process. More studies (Ramkumar et al., 2012; Nguyen-Them et al., 2016; Shao et al., 2020) have shown that PCNSL has a greater relationship with IL-10 and IL-6. Especially IL-10, which is an important biomarker for PCNSL, elevated concentrations of IL-10 in the CSF make PCNSL more aggressive. STAT3 is an important transcription factor and enhanced phosphorylation of STAT is present in late stages of inflammation and immune response, which can promote the expression of immunosuppressive molecules and suppress immune response. It has been shown that elevated STAT3 expression and enhanced phosphorylation in tumour cells of PCNSL can suppress the immune response of tumour cells (Ruggieri et al., 2017; Tang et al., 2021).

Immunosuppressive checkpoints, such as PD-1/PD-L1, are important therapeutic targets in oncology treatment. High expression of PD-1/PD-L1 was present in tumour tissue of PCNSL which was associated with IDO-1 expression (Miyasato et al., 2018; Abdulla et al., 2021). And related studies (Miyasato et al., 2018; Sethi et al., 2019; Furuse et al., 2020) have shown that both macrophages and some T cells surrounding tumour cells have high expression in PD-1, which can promote the suppressive effect of tumour tissues on CD8^+^ T cells. In addition, other checkpoint molecules such as CTLA4, CD86 and TIM3 were also expressed at elevated levels in PCNSL tissues (Alame et al., 2021). Moreover, Biomarkers related with Tumour mutation burden (TMB) were elevated in some PCNSL tissues (Ou et al., 2020). This study showed that there was high expression of immunosuppressive molecules such as PD-L1, CTLA4 and CD86 in the tumour tissue of PCNSL. This suggests that PCNSL can benefit from immunotherapy, while some studies also suggest that PD-1 inhibitors have a better therapeutic effect on relapsed/refractory PCNSL (Lin et al., 2020).

MiRNAs are important protein expression repressors that bind to mRNAs and repress the expression of related proteins. In gliomas and brain tumours, some miRNAs, such as miR16, miR156 and miR21, are in high expression, which can identify the patient’s disease state and even distinguish the category of brain tumours (Ivo D'Urso et al., 2015). More miRNAs are also abnormally expressed in PCNSL tissues. It showed that miR181b, miR30d and miR93 can affect the prognosis of PCNSL patients and are associated with the activation of TGFβ-Notch, MAPK and other pathways (Takashima et al., 2019b). Another studies (Li et al., 2020; Takashima et al., 2020) showed that miR101, miR548b, miR554, miR1202 and miR370 could affect the prognosis of PCNSL patients as potential therapeutic targets.

In recent years, the incidence of PCNSL has increased in line with the rising incidence of autoimmune disease and AIDS. However it is still a lack of effective clinical treatment for PCNSL. The whole brain radiation therapy (WBRT) in combination with chemotherapeutic agents such as methotrexate is still a more common treatment modality. And some studies (Wirsching et al., 2021) have suggested that isitinib may be a more promising therapeutic agent. As immunotherapy becomes more popular, more immune agents such as PD-1/PD-L1 monoclonal antibodies are being used in oncology treatment. This study suggests that inhibition of immunosuppressive checkpoints, such as PD-1/PD-L1,CTLA4,TIM3, may improve the tumour microenvironment in PCNSL. Therefore, immunotherapy may be widely used in the treatment of PCNSL patients in the future. Our study also found that two drugs, OSI.906 and GW.441,756, may have a greater benefit in the treatment of PCNSL, but more studies are needed to verify this result.

## Conclusion

Multi-scale embedded gene co-expression network analysis highlights the crucial role of immune response in the pathophysiological processes of PCNSL. We successfully identify and quantify the molecular characteristics of immunocytes infiltration in PCNSL, which lays the groundwork for improved chemotherapy and immunotherapy for PCNSL. Weighted gene co-expression network analysis identification of key ceRNA network closely associated with immunocytes infiltration score may be promising novel therapeutic targets in PCNSL.

## Data Availability

The datasets presented in this study can be found in online repositories. The names of the repository/repositories and accession number(s) can be found in the article/Supplementary Material.
