# Supplementary figures and images for: Molecular characteristics of immunocytes infiltration in primary central nervous system lymphoma
Source: Front Genet. 2022 Aug 17;13:921823. doi: 10.3389/fgene.2022.921823 (PMC9428130; doi:10.3389/fgene.2022.921823)

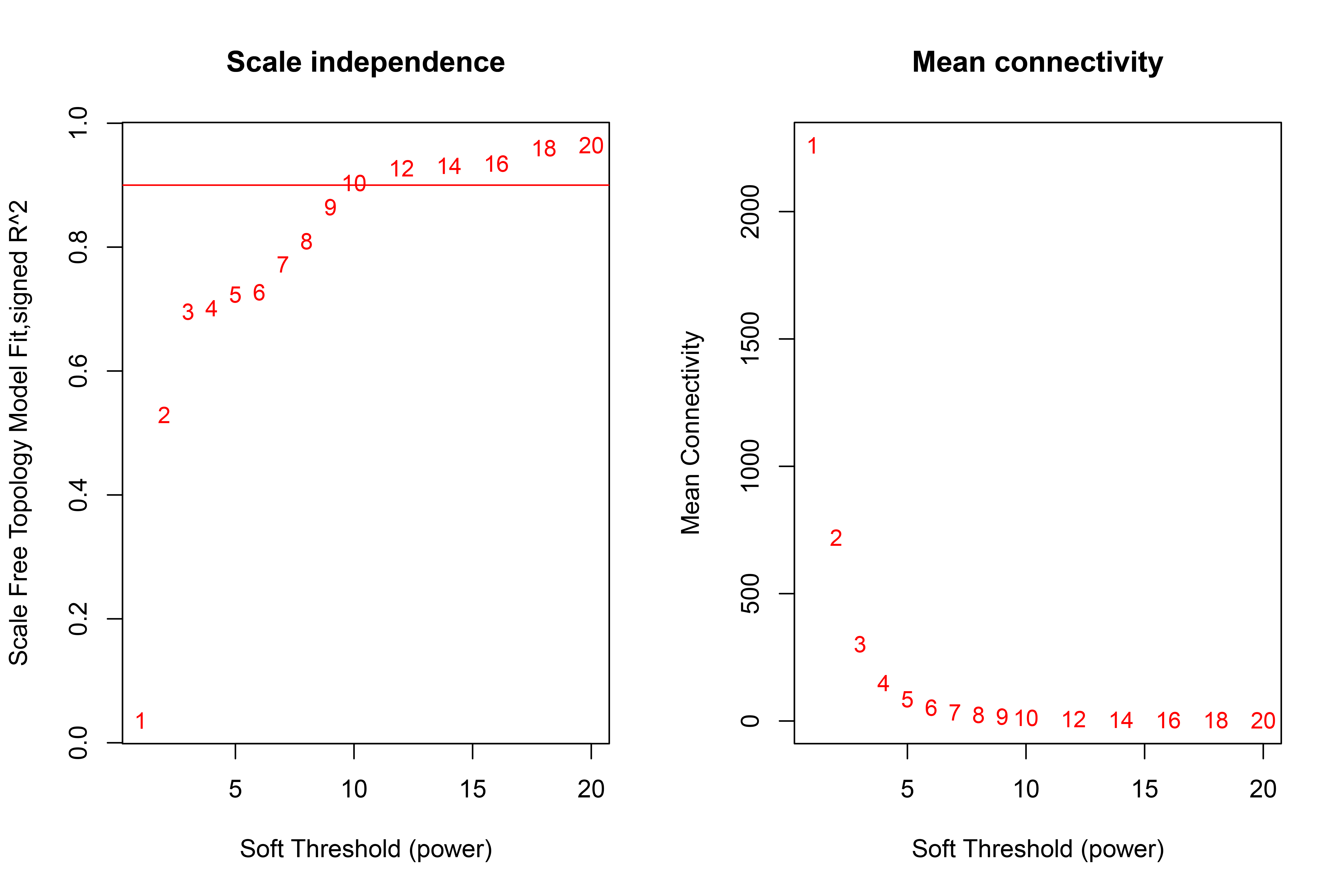

Supplement: Supplementary file 1 [file Image2.JPEG]

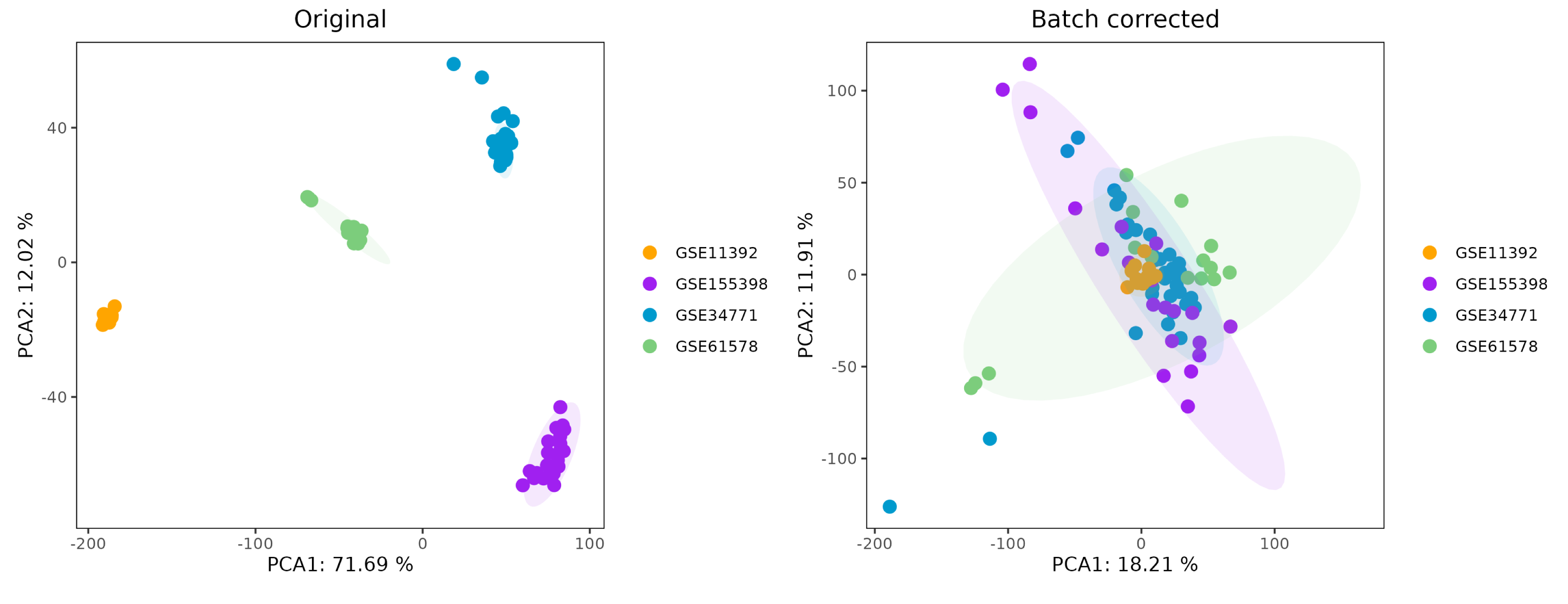

Supplement: Supplementary file 3 [file Image1.PNG]
